# Molecular characterization of *bla*_KHM-1_ encoding plasmid in an *Enterobacter hormaechei* subsp. *hoffmannii* isolate from blood culture

**DOI:** 10.1371/journal.pone.0227605

**Published:** 2020-01-13

**Authors:** Kaoru Umeda, Hiromi Nakamura, Akira Fukuda, Takahiro Yamaguchi, Yuki Matsumoto, Daisuke Motooka, Shota Nakamura, Ryuji Kawahara

**Affiliations:** 1 Division of Microbiology, Osaka Institute of Public Health, Osaka, Japan; 2 Genome Information Research Center, Research Institute for Microbial Diseases, Osaka University, Osaka, Japan; Institut National de la Recherche Agronomique, FRANCE

## Abstract

KHM-1 was first reported in 1997 in Japan as a novel metallo-β-lactamase mediated by *Citrobacter freundii* carrying pKHM-1 plasmid. There have been few reports in the clinical field since then. A *bla*_KHM-1_–positive *Enterobacter hormaechei* subsp. *hoffmannii* in *E*. *cloacae* complex, isolate OIPH-N069 was isolated from an inpatient blood culture in 2016. The isolate was characterized by whole-genome sequencing, comparative analysis of the *bla*_KHM-1_ encoding plasmid, antimicrobial susceptibility tests, and bacterial conjugation. OIPH-N069 was classified into ST78 of *E*. *cloacae* complex, and was multidrug resistant because of the presence of antimicrobial resistance genes in addition to *bla*_KHM-1_ on its chromosome and plasmids. *bla*_KHM-1_ was located on 136,816 bp of the IncA/C_2_ plasmid pN069-1, which could be transferred to different bacterial species. The backbone structure, genetic arrangement of the class 1 integron cassette, and the *bla*_KHM-1_ gene located downstream of the IncA/C_2_ antibiotic resistance island, ARI-A, in pN069-1 and pKHM-1 were identical. Horizontal gene transfer of the *bla*_CTX-M-2_–IS*Ecp1* resistance gene module only occurred with pN069-1. The study findings indicate not only the structural conservation of *bla*_KHM-1_ encoding plasmids over time and across species, but also the risk of the spread of *bla*_KHM-1_ encoding plasmids to other bacterial species and the accumulation of additional resistance genes.

## Introduction

The spread of carbapenemase-producing *Enterobacteriaceae* (CPE) is a serious public health concern [1, 2]. The effectiveness of antibiotics against CPE is limited because carbapenemases can hydrolyze many β-lactams. In addition, CPE isolates are often resistant to other types of antimicrobial agents [2, 3]. The Ambler classification system includes three classes of carbapenemases, class A includes KPC and GES, class B is metallo-β-lactamases including VIM, IMP and NDM, and class D includes OXA-48 [1]. The predominant carbapenemases differ by country and region [1].

KHM-1 was firstly reported as a metallo-β-lactamase produced by *Citrobacter freundii* strain KHM243 isolated in 1997 in a Japanese hospital from a patient with a catheter-associated urinary tract infection [4]. This strain is resistant to most β-lactams other than monobactams. The *bla*_KHM-1_ gene includes a 726-bp open reading frame located on a transferable plasmid and encodes 241 amino acids. The protein has 59% identity with the IMP-1 and SIM-1 metallo-β-lactamases. Since then, there have been few reports on *Enterobacteriaceae* harboring *bla*_KHM-1_ in the clinical field.

Plasmids carrying carbapenemase genes play an important role in the spread of resistance genes to different clones and bacterial species [2]. The plasmids have shown diverse replicon types, and usually have carried carbapenemase genes on a mobile genetic element such as a transposase or insertion sequence [1, 2]. These emphasize the detailed analysis of the plasmids harboring carbapenemase genes can help to understand the mechanism of gene acquisition and to trace the route of transmission.

This study describes an isolate of metallo-beta-lactamase producing and *bla*_KHM-1_–positive *Enterobacter hormaechei* subsp. *hoffmannii* OIPH-N069 isolated from a blood culture in Osaka, Japan in 2016. *E*. *hormaechei* subsp. *hoffmannii* is one of the five subspecies of *E*. *hormaechei* included in *E*. *cloacae* complex [5]. To molecular analysis of this isolates, we performed whole-genome sequencing (WGS), comparative analysis of *bla*_KHM-1_ encoded plasmids, antimicrobial susceptibility tests and bacterial conjugation.

## Materials and methods

### Bacterial isolate

Isolate OIPH-N069 was isolated in 2016 from the blood culture of a hospital inpatient in Osaka, Japan. The isolate was identified as a carbapenem-resistant *Enterobacter cloacae* complex at the hospital laboratory. The biochemical profile was evaluated with an API 20E micro-organism identification kit (Sysmex bioMerieux).

### Antibiotic susceptibility testing

The minimal inhibitory concentrations (MICs) for piperacillin, cefmetazole, cefoxitin, ceftazidime, cefotaxime, cefpodoxime, aztreonam, imipenem, meropenem, gentamicin, amikacin, nalidixic acid, ciprofloxacin, and trimethoprim-sulfamethoxazole were assayed by the Dry Plate EIKEN test (Eiken Chemical). The MIC for fosfomycin (Wako) was evaluated by agar dilution, and the MICs for colistin (Wako), piperacillin/tazobactam (Tokyo Chemical Industry), and tigecycline (Sigma-Aldrich) were assayed by broth dilution according to CLSI document M100-S25 performance standards [6]. *Escherichia coli* ATCC25922 and *Pseudomonas aeruginosa* ATCC27853 were used as controls.

### Phenotypic and genetic analysis of carbapenem resistance

Carbapenemase production was confirmed by the Carba NP test II [7] and double disk synergy test using sodium mercaptoacetic acid (SMA) disks (Eiken Chemical) as an inhibitor. The detection of carbapenemase metallo-β-lactamase genes (*bla*_KHM-1_, *bla*_IMP_, *bla*_VIM_, *bla*_NDM_, *bla*_KPC_, *bla*_OXA-48_, *bla*_SMB,_ and *bla*_GES_) was performed by polymerase chain reaction (PCR) [8].

### Bacterial conjugation

Transfer of the *bla*_KHM-1_ carrying plasmid was confirmed by a filter-mating method described by Kudo [9] with slight modifications. Briefly, the recipient was a rifampicin-resistant *Escherichia coli* K-12 DH5α strain [10]. The donor, recipient, and transconjugant were selected on MacConkey agar (OXOID) supplemented with 0.5 μg/mL meropenem (Wako) and 50 μg/mL rifampicin (Wako) [11]. Colonies were counted after overnight incubation at 37°C. The transfer frequency was reported as the ratio of the numbers of transconjugant to recipient colonies (transconjugant/recipient). The procedure was repeated in triplicate. The transconjugant was tested for antimicrobial sensitivity, and the presence of carbapenemase was confirmed by the CarbaNP test II and PCR of *bla*_KHM-1_ as described above.

### S1-nuclease digested pulsed-field gel electrophoresis (S1-PFGE) and Southern blot hybridization

Agarose gel plugs were prepared by culturing isolates at 37°C for 18 h in trypto-soya broth (Nissui Phamaceutical) and SeaKem Gold agarose (Lonza). Plugs were treated with 1 mg/ml proteinase K (Sigma-Aldrich), digested with 18 U of S1 nuclease (Takara Bio), and electrophoresed on a CHEF-DRIII apparatus (Bio-Rad Laboratories) in 1% SeaKem Gold agarose at 14°C and 6 V/cm for 17 h with switching times of from 2.2 to 54.2 s. The *Salmonella enterica* serovar Braenderup strain H9812 digested with *Xba* I (Roche Diagnostics) was used as a marker for PFGE. DNA fragments were visualized by ethidium bromide staining and then transferred to Hybond N^+^ nylon membranes (GE Healthcare) using a capillary transfer system. Hybridization was carried out with a digoxigenin (DIG)-labeled *bla*_KHM-1_ probe prepared from DNA extracted from isolate OIPH-N069 using a KHM-F/KHM-R primer pair [8] and a PCR DIG probe synthesis kit (Roche Diagnostics). Hybridization signals were detected with a DIG luminescent detection kit (Roche Diagnostics) and an Amersham Imager 600 (GE Healthcare).

### WGS and plasmid sequencing

Genomic DNAs were sheared to 600 bp segments with a focused-ultrasonicator S220 (Covaris) to prepare a short-read sequencing library. DNA was sheared to 8,000 bp segments for long-read sequencing with g-TUBE (Covaris). The short-read library was prepared with dual-indexed, 300 bp paired-end reads using MiSeq Reagent v3 kits (600 cycles) and sequenced with an MiSeq instrument (Illumina). The long-read library was prepared using Ligation Sequencing 1D kits and sequenced with an MinION sequencer (Oxford Nanopore Technologies). Genomic assembly of both short and long reads was performed with Unicycler v0.4.4 [12].

The genome sequences were annotated using the DNA Data Bank of Japan (DDBJ) Fast Annotation and Submission Tool (DFAST, https://dfast.nig.ac.jp/) as described by Tanizawa [13]. In the plasmid analysis, antibiotic resistance genes were identified using ResFinder 3.1 [14], plasmid incompatibility replicon typing was performed with PlasmidFinder 2.0 [15] and multilocus sequence typing (MLST) was performed with MLST 2.0 [16], on the Center for Genomic Epidemiology website (http://www.genomicepidemiology.org/). The bacterial species was identified using the ANI calculator (http://enve-omics.ce.gatech.edu/ani/) [17]. The species identification cut off was 95%–96% of the and the subspecies cutoff was 98% compared with the genomic sequences of the type strain [5, 18, 19]. The insertion sequence element was identified by IS finder (https://www-is.biotoul.fr/index.php). The integron ID was retrieved from the INTEGRALL database (http://integrall.bio.ua.pt/) [20]. Circular representations of plasmid sequences were visualized using the BLAST Ring Image Generator (BRIG, http://brig.sourceforge.net) [21]. The plasmid structure was generated in Easyfig 2.2.3 (https://mjsull.github.io/Easyfig/) [22].

### Nucleotide sequence accession numbers

The complete, annotated genomic sequences were submitted to The DDBJ with the accession numbers AP019817 (chromosome), AP019818 (pN069-1), AP019819 (pN069-2), AP019820 (pN069-3), and AP019821 (pN069-4), associated with BioProject accession number PRJD8177 and Biosample number SAMD00167169.

### Ethical statement

The study was approved by the ethical review committee of Osaka Institute of Public Health (approval No. 1402-04-3).

## Results

### Antimicrobial susceptibilities and detection of *bla*_KHM-1_

The antimicrobial susceptibilities of 18 antibiotics to the OIPH-N069 isolate are shown in Table 1. For β-lactams, this isolate showed resistance to piperacillin, cefmetazole, cefoxitin, ceftazidime, cefotaxime, cefpodoxime, aztreonam and meropenem, and showed intermediate resistance to imipenem and piperacillin/tazobactam. Other, it also showed resistance to trimetoprim-sulpharmethoxazole, nalidixic acid, ciprofloxacin and tigecycline. But, it was susceptible to fosfomycin, aminoglycosides and colistin. The OIPH-N069 isolate was a metallo-β-lactamase producer, positive in both the Carba NP test II and the SMA double disk synergy test. PCR screening of carbapenemase genes detected only *bla*_KHM-1_.

**Table 1:** Antibiotic susceptibility test results.

Antimicrobial agent	MIC (μg/ml) / antimicrobial susceptibility^a^		
	*E*. *hormaechei* subsp. *hoffmannii* OIPH-N069	Transconjugant TcN069	Recipient rifampicin- resitant *E*. *coli* DH5α
Piperacillin	>256 / R	>256 / R	1 / S
Cefmetazole	>64 / R	>64 / R	1 / S
Cefoxitin	>64 / R	>64 / R	4 / S
Ceftazidime	>64 / R	>64 / R	0.125 / S
Cefotaxime	>128 / R	>128 / R	≦0.125 / S
Cefpodoxime	>16 / R	>16 / R	0.5 / S
Aztreonam	16 / R	0.5 / S	0.125 / S
Fosfomycin	32 / S	2 / S	1 / S
Imipenem	2 / I	2 / I	≦0.06 / S
Meropenem	8 / R	8 / R	≦0.06 / S
Gentamicin	0.5 / S	0.5 / S	0.25 / S
Amikacin	1 / S	4 / S	2 / S
Nalidixic Acid	>128 / R	4 / S	4 / S
Ciprofloxacin	16 / R	≦0.03 / S	≦0.03 / S
Trimethoprim-sulphamethoxazole	>4/76 / R	0.12/2.38 / S	0.12/2.38 / S
Colistin	0.25 / S	0.5 / S	0.5 / S
Piperacillin/tazobactam	64/4 / I	4/4 / S	2/4 / S
Tigecycline	1 / R	0.125 / S	0.125 / S

^a^ Breakpoints of *Enterobacteriaceae* generated by CLSI M100-ED29:2019, except for colistin and tigecycline by EUCAST_v9.0.

S, susceptible; I, intermediate; R, resistant.

### Conjugation assay

The conjugation procedure used the OIPH-N069 isolate as the donor and the rifampicin-resistant *E*. *coli* K-12 DH5α strain as the recipient. Transconjugants positive for Carba NP II and *bla*_KHM-1_ by PCR were successfully obtained following selection with meropenem and rifampicin. The antimicrobial susceptibility results obtained with the transconjugant yielded MICs similar or identical to those for the OIPH-N069 isolate, except for lower MICs for aztreonam, fosfomycin, quinolones, trimethoprim-sulfamethoxazole, piperacillin/tazobactam, and tigecycline than those for OIPH-N069 (Table 1). The transfer frequency was 2.9 ×10^−7^.

### S1-PFGE and Southern blot hybridization

**Fig 1:**
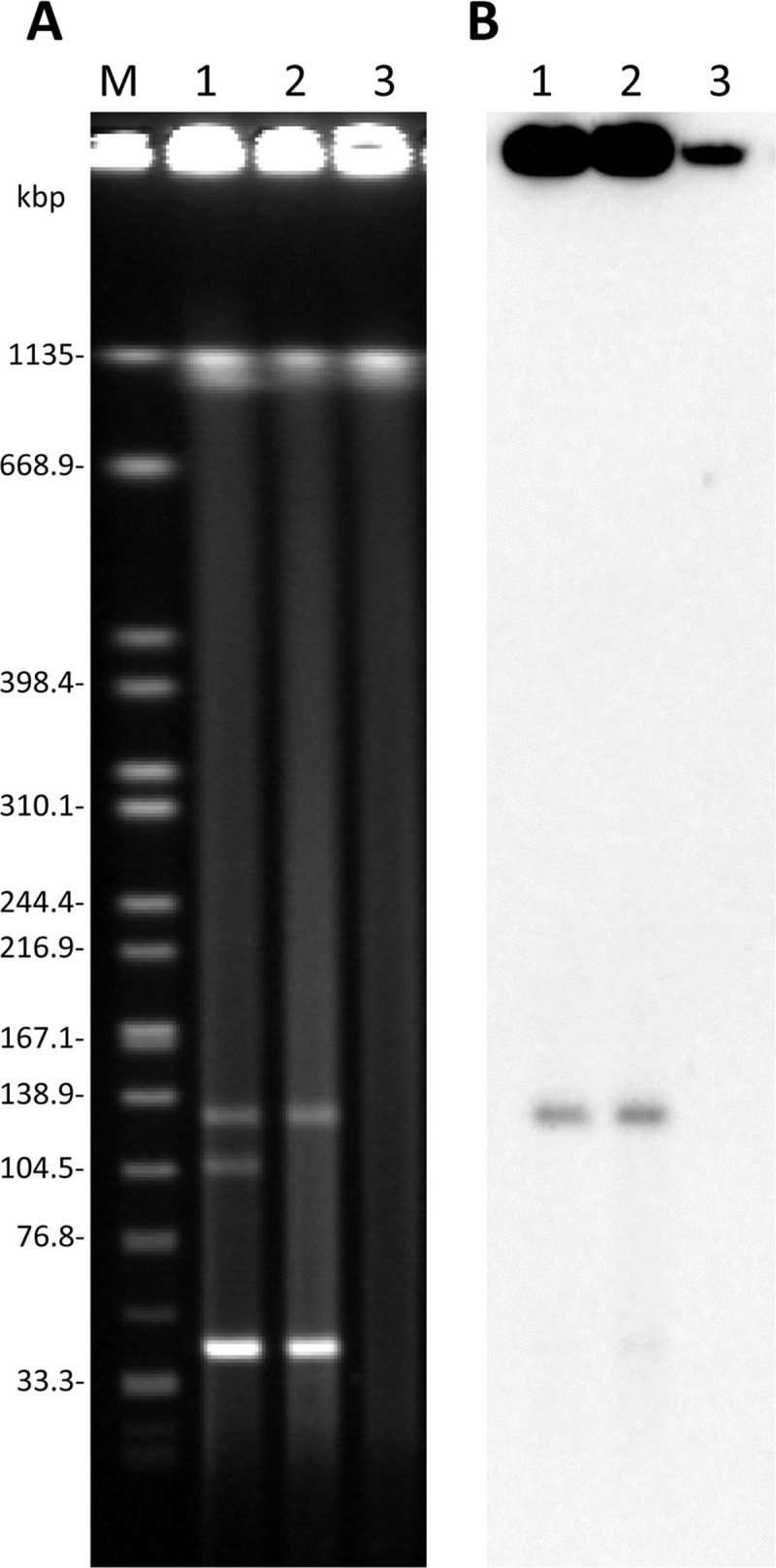
**S1-PFGE (A) and Southern blot hybridization detection (B) of *bla***_**KHM-1**_. Lane 1, *E*. *hormaechei* subsp. *hoffmannii* isolate OIPH-N069; Lane 2, transconjugant TcN069; Lane 3, *E*. *coli* K-12 DH5α (recipient); Lane M; *Xba* I-digested *Salmonella enterica* serovar Braenderup strain H9812.

The S1-PFGE results showed that the OIPH-N069 isolate carried three plasmids in addition to the bacterial chromosome. The TcN069 transconjugant carried two plasmids from the OIPH-N069 isolate (Fig 1A). Southern blot hybridization found that *bla*_KHM-1_ was located on a plasmid with approximately130 kbp in both the OIPH-N069 isolate and the TcN069 transconjugant (Fig 1B).

### WGS analysis of the OIPH-N069 isolate

The genome sequences of the OIPH-N069 isolate are shown in Table 2. The OIPH-N069 genome comprised a chromosome and four plasmids and carried nine antimicrobial resistance genes. The genome included chromosomal sequences that incuded 4,689,117 bp identified as *E*. *hormaechei* subsp. *hoffmannii* by the average nucleotide identity (ANI) value compared with the genomic sequences of the type strains of the *E*. *cloacae* complex [5, 18]. The biochemical profile of the OIPH-N069 isolate was identical to that of *E*. *cloacae* complex. The sequence type (ST) for the chromosome was ST78. The chromosome encoded antimicrobial resistance genes of *bla*_ACT-5_ and *fosA*, and also had mutations in quinolone resistance-determining regions (QRDR) of *gyr*A (Ser/Thr83 to Ile) and *par*C (Ser80 to Ile).

**Table 2:** Whole genome information for *E*. *hormaechei* subsp. *hoffmannii* isolate OIPH-N069.

Replicon	Nulceotide length (bp)	Number of coding sequence	GC%	Inc type	Antimicrobial resistance genes	Accession No.
Chromosome	4,689,117	4,375	55.2	NA	*bla*_ACT-5_, *fosA*	AP019817
pN069-1	136,816	163	50.1	IncA/C_2_	*aadA2*, *aac(6')-lae*, *bla*_CTX-M-2_, *bla*_KHM-1_, *sul1*	AP019818
pN069-2	115,150	139	51.8	IncFIB	*dfrA15*, *sul1*	AP019819
pN069-3	47,299	63	51.1	NA	ND	AP019820
pN069-4	2,495	3	51.5	ColRNAI	ND	AP019821

NA, not available; ND, not detected.

The four plasmids included pN069-1 (136,816bp, IncA/C_2_, harbored *aadA2*, *aac(6’)-lae*, *bla*_CTX-M-2_, *bla*_KHM-1_ and *sul1*), pN069-2 (115,150 bp, IncFIB, harbored *dfrA15* and *sul1*), pN069-3 (47,299 bp, Inc type was not available) and pN069-4 (2,495 bp, ColRNAI plasmid).

The lower MICs of transconjugant TcN069 than isolate OIPH-N069 for aztreonam, quinolones, fosfomycin, and trimethoprim-sulphamethoxazole could relate to *bla*_ACT-5_ on chromosome, QRDR mutations on chromosome, *fosA* on chromosome, and *drfA* on pN069-2, respectively.

### Comparative analysis of *bla*_KHM-1_ on pN069-1

**Fig 2:**
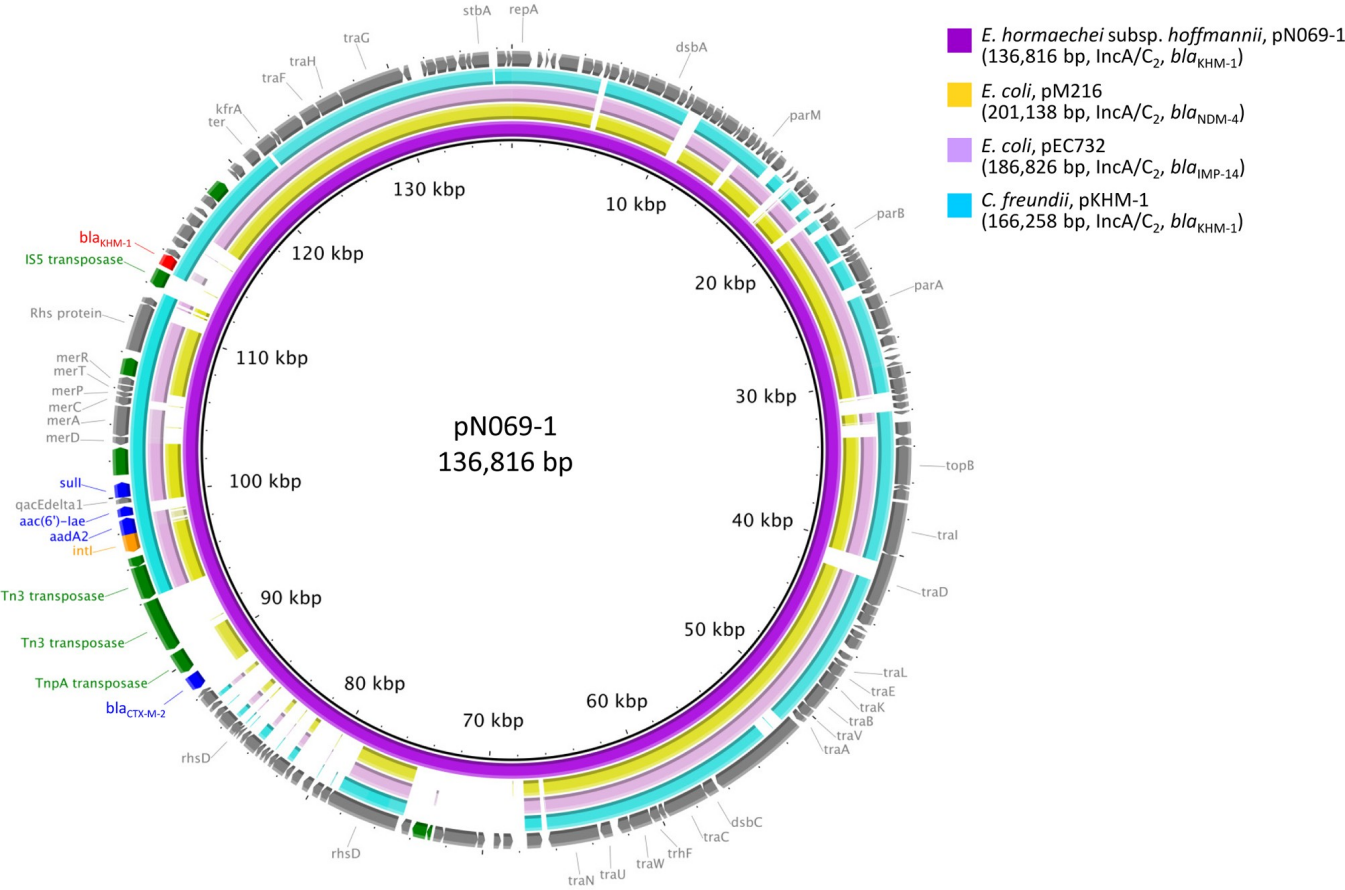
Circular representation of pN069-1. The comparison of pN069-1 and three plasmids; pKHM-1 from *C*. *freundii* strain KHM243 (AP014939) [4], pEC732 from *E*. *coli* (CP015139) [23] and pM216 from *E*. *coli* (AP018145) [24]. The outermost circle shows the coding sequence of pN069-1. Red, *bla*_KHM-1_; blue, other antimicrobial resistance genes; green, transposase and recombinase genes; orange, integrase genes; gray, other genes or coding sequences.

The results of comparative genomic analysis of *bla*_KHM-1_ encoded on plasmid pN069-1 and on pKHM-1, pM216, and pEC732 are shown in Fig 2. The pKHM-1 from *C*. *freundii* strain KHM243 (AP014939) [4] was identified as an IncA/C_2_ type by PlasmidFinder 2.0. The other IncA/C_2_ plasmids, pEC732 (74% query cover and 94.41% identity) carrying *bla*_IMP-14_ from *E*. *coli* (CP015139) [23] and pM216 (73% query cover and 94.41% identity) carrying *bla*_NDM-4_ from *E*. *coli* (AP018145) [24], were selected by their homology score following a BLAST search using the whole nucleotide sequence of pN069-1. The backbone structure of pN069-1 was conserved in pKHM-1 and the two other IncA/C_2_ plasmids, but variation of the antimicrobic-resistance genes and their surrounding regions was observed. In pN069-1, the *bla*_KHM-1_ gene was located about 12.3 kbp downstream of the class 1 integron cassette. *bla*_KHM-1_ and its adjacent structure were present in pN069-1 and pKHM-1 only. *bla*_CTX-M-2_ and its adjacent genes containing transposases were only found in pN069-1.

### Genetic structure of antimicrobial resistance regions

**Fig 3:**
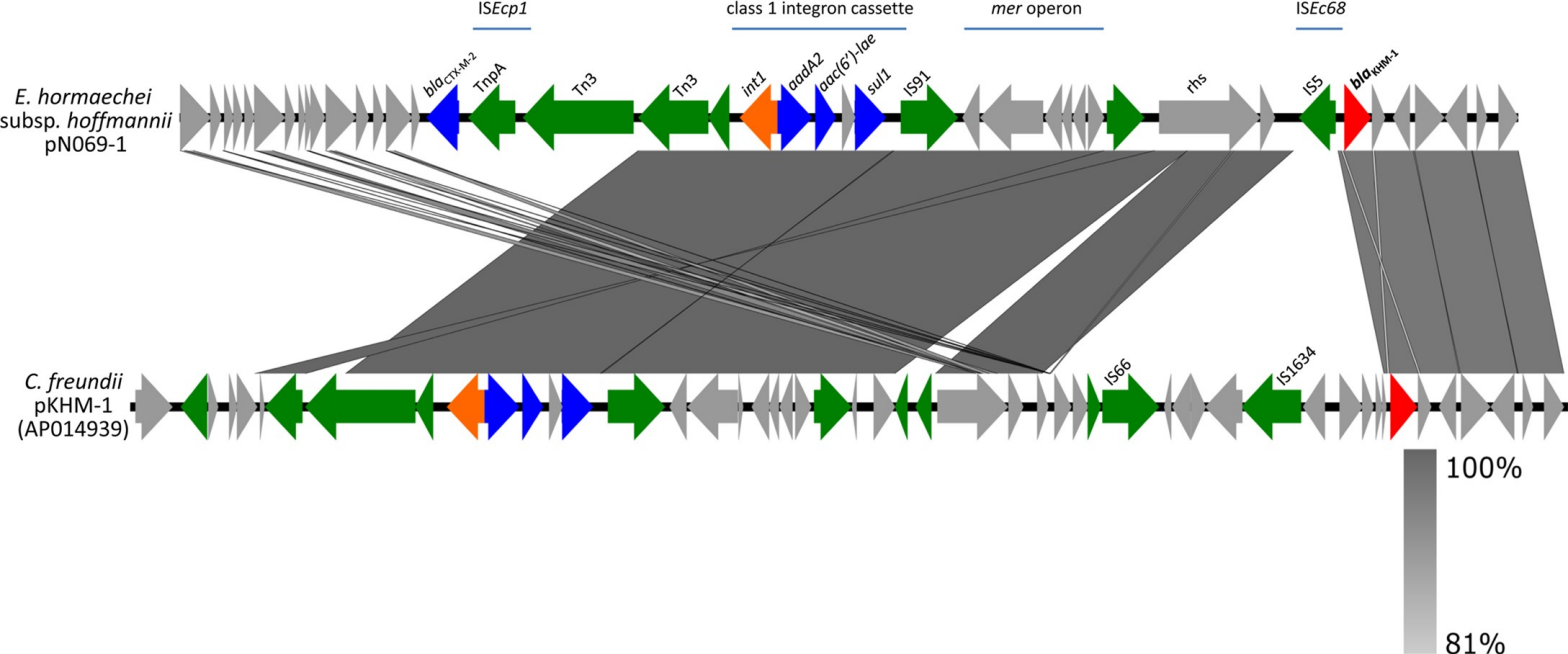
Genetic structures of antimicrobial resistance regions. The *bla*_KHM-1_ surrounding antimicrobial resistance region of pN069-1 (about 36.1 kbp) was aligned with that of pKHM-1 (AP014939) [4]. Red, *bla*_KHM-1_; blue, other antimicrobial resistance genes; green, transposase and recombinase genes; orange, integrase genes; gray, other genes or coding sequences.

The genetic structure of the antimicrobial resistance region in pN069-1 was compared with that in pKHM-1 (Fig 3). This region contained several transposase/recombinase genes. The *bla*_KHM-1_ nucleotide sequences (100% identity) and the gene arrangements of its downstream containing lyoxalase family and hypothetical proteins were well conserved in both plasmids. The class 1 integron cassette, its surrounding transposases containing Tn3 and IS91 family protein genes, and the *mer*-operon were also homologous in both plasmids. The genetic structure of class 1 integron cassette [*int1*, *aadA2*, *aac(6)-lae*, *qacEdelta1*, and *sul1*] was not found in GenBank or the INTEGRALL database. The IS5 family transposase (identified as IS*Ec68*) located just upstream of *bla*_KHM-1_, *bla*_CTX-M-2_ with the tnpA family transposase (identified as IS*Ecp1*) were found only in pN069-1. Fourteen coding sequences containing IS66 and IS1634 family transposases located approximately 9.2 kbp upstream of *bla*_KHM-1_ were found only in pKHM-1.

## Discussion

In this study, we analyzed a KHM-1-producing *E*. *hormaechei* subsp. *hoffmannii* isolate OIPH-N069 from the blood sample of an inpatient. *E*. *hormaechei* is the most frequently identified in clinical isolates of *E*. *cloacae* complex member [5], and ST78 is one of the most common clone among multidrug- or carbapenem-resistant isolates in worldwide [25] including in Japan [26]. OIPH-N069 was isolated 19 years after the *C*. *freundii* KHM243 strain [4], but the backbone structure of plasmids and peripheral structure of *bla*_KHM-1_ were relatively well conserved, even with the presence of some deletions and insertions (Figs 2 and 3). Although the *bla*_KHM-1_ encoding plasmid has rarely found, it may have been repeatedly transferred to multiple bacterial species and maintained for many years. The results of conjugation assay (Fig 1) also suggest that the *bla*_KHM-1_ encoding IncA/C_2_ plasmid can spread to other species. Just recently, KHM-1–producing *Klebsiella quasipneumoniae* subsp. *quasipneumoniae* was isolated from wastewater in Tokyo, Japan [27]. Ongoing comprehensive surveillance with characterization of antimicrobial resistance plasmids is recommended to monitor the spread and resistance of KHM-1–producing *Enterobacteriaceae* in clinical and other settings.

OIPH-N069 showed resistance to several kinds of antimicrobials, and that corresponded to antimicrobial resistant genes on its chromosome and plasmids (Tables 1 and 2). OIPH-N069 was found to be a multidrug-resistant *Enterobacteriaceae* according to the international standard criteria described by Magiorakos et al. [28], and more resistant than *C*. *freundii* KHM243 such as aztreonam and tigecycline, which are used to treat infections caused by carbapenem-resistant *Enterobacteriaceae* [3, 4] (Table 1).

The antibiotic resistance island of the IncA/C_2_ plasmid located upstream of the *rhs* gene is called as the ARI-A region [29, 30], but *bla*_KHM-1_ was located downstream of the ARI-A island, both in pN069-1 and pKHM-1 (Figs 2 and 3). The IS5 family transposase IS*Ec68* was found just upstream of *bla*_KHM-1_ in pN069-1, but pKHM-1 had another transposases. So that, the origin of *bla*_KHM-1_ and how it came to be inserted in this region could not be determined. The class 1 integron cassette in the pN069-1 harbored resistance genes of *aadA2*, *aac(6’)-lae* and *sul1*, homologous with pKHM-1, and also the other IncA/C_2_ plasmids except for *aac(6’)-lae*. The *bla*_CTX-M-2_ was present only in the ARI-A region of pN069-1 (Figs 2 and 3). The *bla*_CTX-M-2_-IS*Ecp1* module has been reported in antimicrobial resistance plasmids from other species, for example, the multidrug resistance IncN plasmid pKPI-6 from *Klebsiella pneumoniae* (AB616660) epidemic in Japan [31]. It suggests the horizontal acquisition of IS*Ecp1*-mediated *bla*_CTX-M-2_ as a new resistance gene in pN069-1. As the ARI-A region of the IncA/C_2_ plasmid is a reservoir of resistance genes [29, 30], the *bla*_KHM-1–_encoding plasmid may become increasingly resistant in the future.

In conclusion, molecular characterization and WGS of an *E*. *hormaechei* subsp. *hoffmannii* ST78 isolate producing KHM-1 metallo-β-lactamase confirmed not only the structural conservation of *bla*_KHM-1–_encoding plasmids over time and across species, but also that the *bla*_KHM-1–_encoding plasmid can spread to other *Enterobacteriaceae* species and horizontally acquire microbial resistance genes. In the clinical aspect, antimicrobial treatment of infections caused by KHM-1-producing *Enterobacteriaceae* must be careful due to the possibility for MDR with multiple resistant genes in addition to *bla*_KHM-1_.

## Supporting information

S1 Fig Original images for gel and blot. S1-PFGE gel image for Fig 1A and Southern blot hybridization image for Fig 1B were represented.
(PDF)

## References

[1] Logan LK, Weinstein RA. The epidemiology of carbapenem-resistant *Enterobacteriaceae*: the impact and evolution of a global menace. J Infect Dis. 2017; 215:S28–S36. 10.1093/infdis/jiw282 28375512 PMC5853342

[2] van Duin D, Doi Y. The global epidemiology of carbapenemase-producing *Enterobacteriaceae*. Virulence. 2017; 8:460–469. 10.1080/21505594.2016.1222343 27593176 PMC5477705

[3] Sheu C, Chang Y, Lin S, Chen Y, Hsueh P. Infections caused by carbapenem-resistant *Enterobacteriaceae*: an update on therapeutic options. Front Microbiol. 2019; 10:80 10.3389/fmicb.2019.00080 30761114 PMC6363665

[4] Sekiguchi J, Morita K, Kitao T, Watanabe N, Okazaki M, Miyoshi-Akiyama T, et al KHM-1, a novel plasmid-mediated metallo-β-lactamase from a *Citrobacter freundii* clinical isolate. Atimicrob Agents Chemother. 2008; 52:4194–4197. 10.1128/AAC.01337-07 PMC2573105 18765691

[5] Sutton G, Brinkac L, Clarke T, Fouts D. *Enterobacter hormaechei* subsp. *hoffmannii* subsp. nov., *Enterobacter hormaechei* subsp. *xiangfangensis* comb. nov., *Enterobacter roggenkampii* sp. nov., and *Enterobacter muelleri* is a later heterotypic synonym of *Enterobacter asburiae* based on computational analysis of sequenced *Enterobacter* genomes. F1000Res. 2018; 7:521 10.12688/f1000research.14566.2 30430006 PMC6097438

[6] Clinical and Laboratory Standard Institute. Performance standards for antimicrobial susceptibility testing: Twenty-fifth informational supplement, CLSI document M100-S25 Wayne, PA Clinical Laboratory Standards Institute 2015.

[7] Dortret L, Poirel L, Nordmann P. Rapid identification of Carbapenemase types in *Enterobacteriaceae* and *Pseudomonas* spp. by using a biochemical test. Antimicrob Agents Chemother. 2012; 56:6437–6440. 10.1128/AAC.01395-12 23070158 PMC3497194

[8] National Institute of Infectious Diseases. Pathogen detection manual, drug resistant bacteria (in Japanese). 2016. Available from:https://www.niid.go.jp/niid/images/lab-manual/ResistantBacteria201612V1.1.pdf.

[9] Kudo H, Usui M, Nagafuji W, Oka K, Takahashi M, Yamaguchi H, et al Inhibition effect of flavophospholipol on conjugative transfer of the extended-spectrum β-lactamase and *van*A genes. J Antibiot. 2019; 72:79–85. 10.1038/s41429-018-0113-4 30361635 PMC6760635

[10] Usui M, Hiki M, Murakami K, Ozawa M, Nagai H, Asai T. Evaluation of transferability of R-plasmid in bacteriocin-producing donors to bacteriocin-resistant recipients. Jpn J Infect Dis. 2012; 65:252–255. 10.7883/yoken.65.252 22627309

[11] Jin Y, Song X, Liu Y, Wang Y, Zhang B, Fan H, et al Characteristics of carbapenemase-producing *Klebsiella pneumoniae* as a cause of neonatal infection in Shandong, China. Exp Ther Med. 2017; 13:1117–1126. 10.3892/etm.2017.4070 28450951 PMC5403258

[12] Wick R, Judd M, Gorrie C, Holt K. Unicycler: Resolving bacterial genome assemblies from short and long sequencing reads. PLoS Comput Biol. 2017; 13:e1005595 10.1371/journal.pcbi.1005595 28594827 PMC5481147

[13] Tanizawa Y, Fujisawa T, Nakamura Y. DFAST: a flexible prokaryotic genome annotation pipeline for faster genome publication. Bioinformatics. 2018; 34:1037–1039. 10.1093/bioinformatics/btx713 29106469 PMC5860143

[14] Zankari E, Hasman H, Cosentino S, Vestergaard M, Rasmussen S, Lund O, et al Identification of acquired antimicrobial resistance genes. J Antimicrob Chemother. 2012; 67:2640–2644. 10.1093/jac/dks261 22782487 PMC3468078

[15] Carattoli A, Zankari E, Garcia-Fernandez A, Voldby Larsen M, Lund O, Villa L, et al In silico detection and typing of plasmids using PlasmidFinder and plasmid multilocus sequence typing. Antimicrob Agents Chemother. 2014; 58:3895–3903. 10.1128/AAC.02412-14 24777092 PMC4068535

[16] Larsen M, Cosentino S, Rasmussen S, Friis C, Hasman H, Marvig R, et al Multilocus sequence typing of total genome sequenced bacteria. J Clin Microbiol. 2012; 50:1355–1361. 10.1128/JCM.06094-11 22238442 PMC3318499

[17] Goris J, Konstantinidis K, Klappenbach J, Coenye T, Vandamme P, Tiedje J. DNA-DNA hybridization values and their relationship to whole-genome sequence similarities. Int J Syst Evol Microbiol. 2007; 57:81–91. 10.1099/ijs.0.64483-0 17220447

[18] Chavda K, Chen L, Fouts D, Sutton G, Brinkac L, Jenkins S, et al Comprehensive genome analysis of carbapenemase-producing *Enterobacter* spp.: New insights into phylogeny, population structure, and resistance mechanisms. MBio. 2016; 7:e02093–e02016. 10.1128/mBio.02093-16 27965456 PMC5156309

[19] Kim M, Oh H, Park S, Chun J. Towards a taxonomic coherence between average nucleotide identity and 16S rRNA gene sequence similarity for species demarcation of prokaryotes. Int J Syst Evol Microbiol. 2014; 64:346–351. 10.1099/ijs.0.059774-0 24505072

[20] Moura A, Soares M, Pereira C, Leitão N, Henriques I, Correia A. INTEGRALL: a database and search engine for integrons, integrases, and gene cassettes. Bioinformatics. 2009; 25:1096–1098. 10.1093/bioinformatics/btp105 19228805

[21] Alikhan N, Petty N, Ben Zakour N, Beatson S. BLAST Ring Image Generator (BRIG): simple prokaryote genome comparisons. BMC Genomics. 2011; 12:402 10.1186/1471-2164-12-402 21824423 PMC3163573

[22] Sullivan M, Petty N, Beatson S. Easyfig: a genome comparison visualizer. Bioinformatics. 2011; 27:1009–1010. 10.1093/bioinformatics/btr039 21278367 PMC3065679

[23] Stoesser N, Sheppard A, Peirano G, Sebra R, Lynch T, Anson L, et al First report of *bla*_IMP-14_ on a plasmid harboring multiple drug resistance genes in *Escherichia coli* sequence type 131. Antimicrob Agents Chemother. 2016; 60:5068–5071. 10.1128/AAC.00840-16 27246777 PMC4958194

[24] Sunagawa Y, Akeda Y, Sakamoto N, Takeuchi D, Motooka D, Nakamura S, et al Genetic characterization of *bla*_NDM_-harboring plasmids in carbapenem-resistant *Escherichia coli* from Myanmer. PLoS ONE. 2017; 12:e0184720 10.1371/journal.pone.0184720 28910381 PMC5598989

[25] Annavajhala M, Gomez-Simmonds A, Uhlemann A. Multidrug-resistant *Enterobacter cloacae* complex emerging as a grobal, dibersifying threat. Front Microbiol. 2019; 10:44 10.3389/fmicb.2019.00044 30766518 PMC6365427

[26] Aoki K, Harada S, Yahara K, Ishii Y, Motooka D, Nakamura S, et al Molecular characterization of IMP-1-producing *Enterobacter cloacae* complex isolates in Tokyo. J Antimicrob Chemother. 2018; 62:e02091–17. 10.1128/AAC.02091-17 PMC5826111 29311089

[27] Suzuki Y, Ida M, Kubota H, Ariyoshi T, Murakami K, Kobayashi M, et al Multiple β-lactam resistance gene-carrying plasmid harbored by *Klebsiella quasipneumoniae* isolated from urban sewage in Japan. mSphere. 2019; 4:e00391–19. 10.1128/mSphere.00391-19 31554719 PMC6763765

[28] Magiorakos A, Srinivasan A, Carey R, Carmeli Y, Falagas M, Giske C, et al Multidrug-resistant, extensively drug-resistant and pandrug-resistant bacteria: an international expert proposal for interim standard definitions for acquired resistance. Clin Microbiol Infect. 2011; 18:268–281. 10.1111/j.1469-0691.2011.03570.x 21793988

[29] Harmer C, Hall R. The A to Z of A/C plasmids. Plasmid. 2015; 80:63–82. 10.1016/j.plasmid.2015.04.003 25910948

[30] Papagiannitsis C, Dolegjska M, Izdebski R, Giakkoupi P, Skalova A, Chudějová K, et al Characterization of IncA/C2 plasmids carrying an In416-like integron with the *bla*_VIM-19_ gene from *Klebsiella pneumoniae* ST383 of Greek origin. Int J Antimicrob Agents. 2016; 47:158–162. 10.1016/j.ijantimicag.2015.12.001 26795022

[31] Kayama S, Shigemoto N, Kuwahara R, Oshima K, Hirakawa H, Hisatsune J, et al Complete nucleotide sequence of the IncN plasmid encoding IMP-6 and CTX-M-2 from emerging carbapenem-resistant *Enterobacteriaceae* in Japan. Antimicrob Agents Chemother. 2015; 59:1356–1359. 10.1128/AAC.04759-14 25487806 PMC4335907

