# Supplementary material for: Molecular characterization of blaKHM-1 encoding plasmid in an Enterobacter hormaechei subsp. hoffmannii isolate from blood culture
Source: PLoS One. 2020 Jan 13;15(1):e0227605. doi: 10.1371/journal.pone.0227605 (PMC6957179; doi:10.1371/journal.pone.0227605)

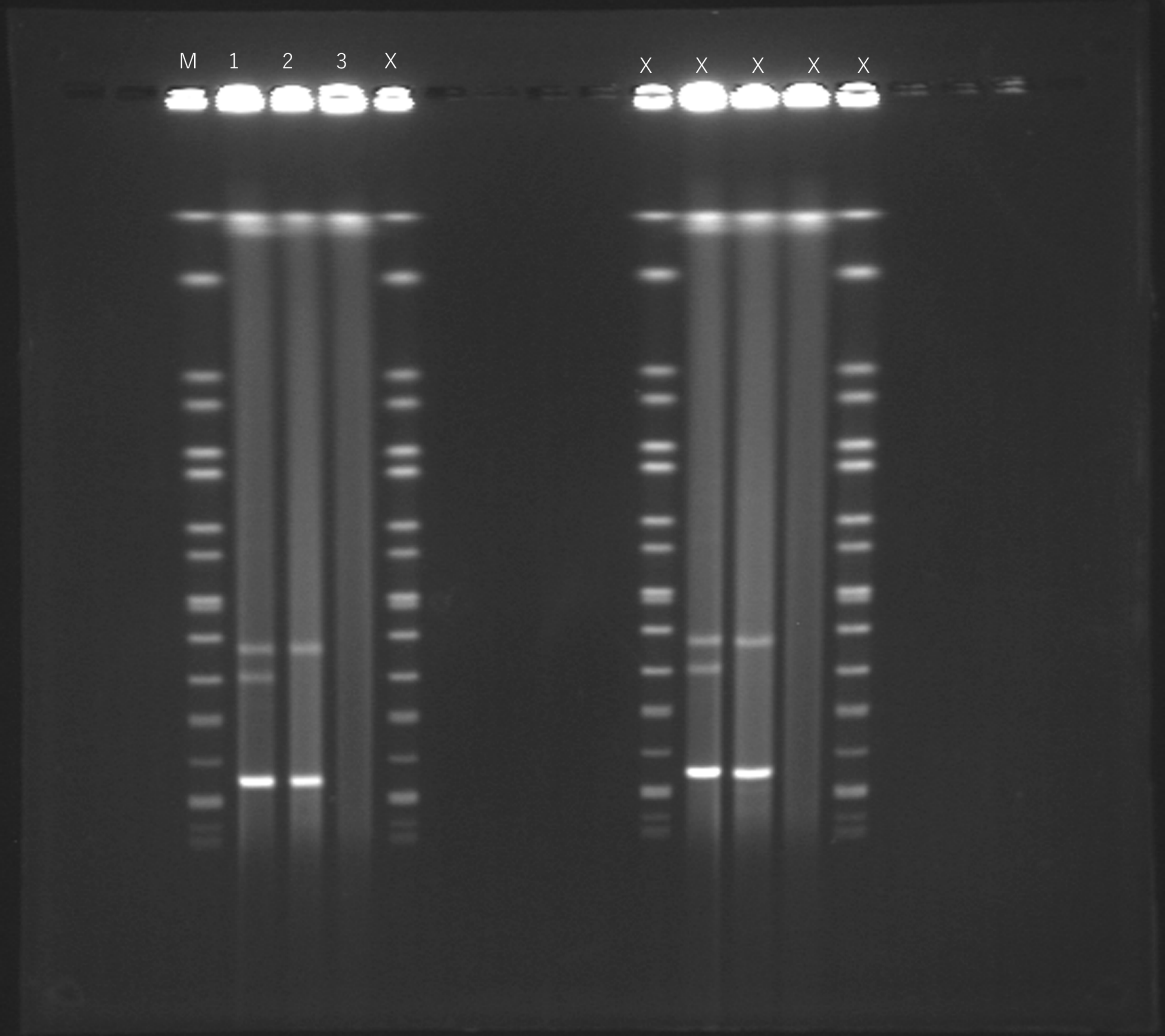

Lanes M and 1 to 3 corresponded to Fig 1A of Lanes M and 1 to 3. SI-PFGE were dupricated in the same gel, and only left side lanes were used as Fig. 1A.DNA fragments were visualized using Bio-Doc It Imaging System (UVP).

Lanes 1 to 3 correspond to Lanes 1 to 3 in Fig 1B.

Left side 5 lanes of Fig 1A. raw image were transfarred to Hybond N+membrane, and Southern blot hybridization were performed with DIG-labeled KHM-1 probe. Hybridization signals were ditected using Amaesham Imager 600 (GE Healthcare).

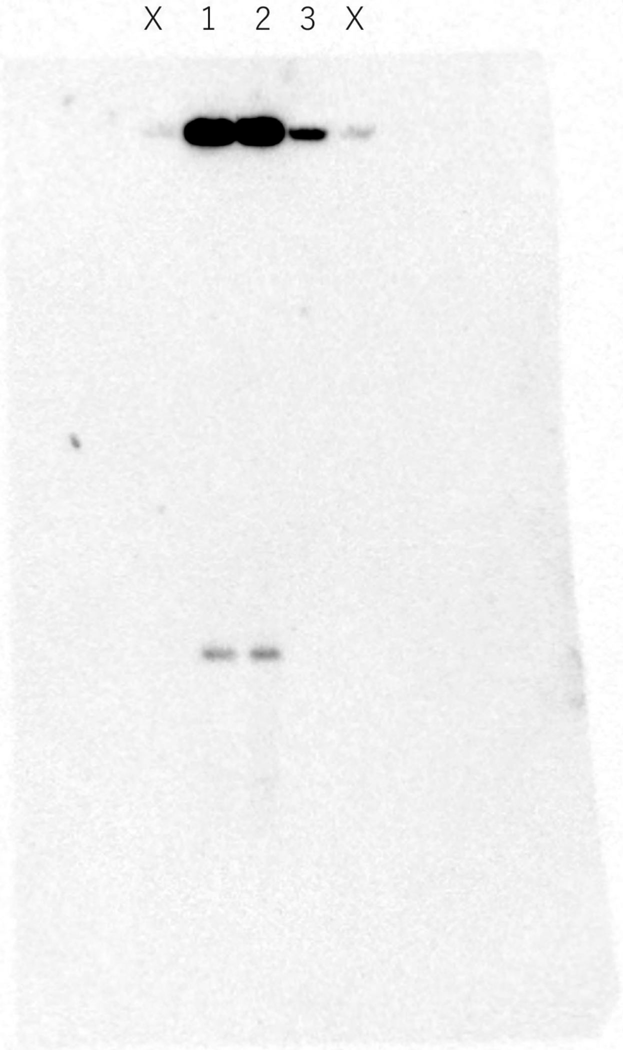

Supplement: S1 Fig — S1-PFGE gel image for Fig 1A and Southern blot hybridization image for Fig 1B were represented. (PDF) [file pone.0227605.s001.pdf]
